# Editorial: Role of metal ions in central nervous system: Physiology and pathophysiology

**DOI:** 10.3389/fncel.2022.1093224

**Published:** 2022-11-22

**Authors:** Feng Guo, Ludmila Zylinska, Tomasz Boczek

**Affiliations:** ^1^Department of Pharmaceutical Toxicology, China Medical University, Shenyang, China; ^2^Department of Molecular Neurochemistry, Medical University of Lodz, Lodz, Poland

**Keywords:** neurodegenerating diseases, metal ions, retina, pain sensation, Parkinson's disease, cAMP signaling, astrocytes, osmoregulation

Maintenance of ion gradient across the plasma membrane is of paramount importance for neuronal physiology. The loss of control over these gradients may have severe consequences ultimately leading to neuronal death, premature aging and variety of public health diseases, including Parkinson's disease, or nerve tumors. Metal ions are, in particular, closely related to the proper function of the central nervous system. On the other hand, their dyshomeostasis may cause neurotoxicity and contribute to the development of pathological states and therefore, mechanisms regulating intra- and extracellular ion balance are important pharmacological targets in numerous neurological diseases. Despite the recent progress, ongoing initiatives are needed for deep understanding of early ion imbalance-related pathogenic changes for development of successful treatment strategies. This Research Topic consists of four original papers and two reviews providing readers of the journal with recent discoveries related to dysfunctions of metal homeostasis in the central nervous system pathology.

Ca^2+^ plays a pivotal role in neuronal differentiation and specialization. Imbalance in Ca^2+^ homeostasis may have detrimental consequences for newborn neurons survival and lead to massive neuronal loss. During embryonal stage, neuronal development is strongly affected by psychomimetic drugs including ketamine. Lisek et al. demonstrated that heterologous expression of plasma membrane Ca^2+^-ATPase isoform 2b (PMCA2b) in hippocampal progenitor cells accelerated basic fibroblast growth factor (bFGF)-dependent differenation and protected from ketamine-induced death. The protection involved neither changes in a resting Ca^2+^ concentration nor calcium clearing potency. The authors suggested complex rescue adaptations due to compensatory up-regulation of N-methyl-D-aspartate receptor subunit 1 and its enhanced co-immunoprecipitation with PMCA2b. Besides that, they demonstrated the expression change of several genes encoding proteins key to GABA metabolism. The presence of PMCA2b normalized deficient Ca^2+^-induced GABA release demonstrated in the presence of ketamine. As a putative mechanism, the authors proposed a shift of GABA utilization from energetic purposes toward neurosecretion, based on the observation of PMCA2b-dependnet inhibition of GABA transaminase. This study constitutes the first experimental evidence showing that developmentally-controlled PMCA expression is crucial for differentiation of hippocampal progenitor cells.

The review paper by Sobolczyk et al. aimed to discuss the crosstalk between Ca^2+^ and cAMP-dependent signaling in astrocytes in the context of neurodegeneration. The results present a comprehensive analysis of G protein-coupled receptor-dependent and independent effects on both second messenger systems in a brain-region specific manner and plausible consequences on gliotransmission. Moreover, the authors investigated how altered Ca^2+^/cAMP signaling is involved in glutamate excitotoxicity and thoroughly analyzed its relationship to neurotrophic support. Based on the experimental data, they also pointed out the idea that disrupted coupling between Ca^2+^/cAMP signaling, astrocytic aquaporin-4 (AQP4)-dependent water permeability and apolipoprotein E role may be imported for pathophysiology of neurogenerative diseases. It was concluded that local brain dynamics in astrocytes may determine the phenotype of synaptic damage and axonal loss.

The second review by Xu et al. focused on the role of Ca^2+^-dependent neurodegeneration in Parkinson's disease. The authors first discussed how neuronal Ca^2+^ imbalance may affect the crosstalk between endoplasmic reticulum, mitochondria and lysosomes promoting structural and functional alterations of dopaminergic neurons in the substantia nigra pars compacta. Further, they expanded their discussion on several other pathological changes including SNCA, GBA, LRRK2, PRKN and PINK1 gene mutations, environmental toxins or head trauma, which are known to exacerbate neuronal Ca^2+^ dyshomeostasis. The authors indicated that restoration of Ca^2+^ balance in cellular organelles may turn out to be a promising novel strategy for Parkinson's disease treatment.

Abnormal copper homeostasis has repeatedly been confirmed to affect many key cellular processes and play important role in nerve tumor progression. Wang X. et al. performed an analysis of copper-related protein expression in glioma patients and selected 90 potential targets from the Cancer Genome Atlas (TCGA) and REMBRANDT databases. Based on prognostic value of these differentially-expressed proteins, they developed a model to show that three genes (LOXL3, ANG and STEAP2) significantly affected the overall survival of patients with glioma but only increased expression of STEAP2 correlated with longer survival of patients with glioblastoma multiforme. They also identified the tumor mutational burden of copper-related proteins and discussed the differences in the immune microenvironment in the high-risk and low-risk groups. Finally, they emphasized the need of a further research on copper-related proteins as promising biomarkers in clinical applications.

Marshall and Crewther employed elemental microanalysis of light-adapted frozen hydrated chick eyes to quantitatively characterize the pattern of biological elements as well as hydration state across retina layers. Trace elements calcium, zinc, iron, potassium, barium and copper have been localized showing more complex osmoregulatory functions within the normal retina, which underlines the ionic control of neuronal processing and fluid movements during light-dark modulation. Calculated osmotic gradients supported the hypothesis about increasing gradient and water flow across the inner retina to the photoreceptor/apical retinal pigment epithelium. Adding taurine to the calculations suggested more increased osmotic water transport from the vitreous into the ganglion cell layers indicating that water crosses the membrane from the retina into the retinal pigment epithelium down the Na^+^ and K^+^ gradients. This clinically important data improve the models of retinal fluid dynamics and help to understand the molecular mechanisms of myopia.

Understanding of molecular mechanisms for pain sensation is crucial for effective development of novel analgesics. Two major non-overlapping subpopulations of C-fiber nociceptors have been identified and their activation has been reported to provoke diverse nocifensive behaviors. Using transgenic mice expressing channel rhodopsin in a subpopulation of the Mas-related G protein-coupled receptor D (Mrgprd^+^) or transient receptor potential vanilloid 1 (TRPV1^+^) sensory neurons, Wang L.-B. et al. demonstrated that Mrgprd^+^ nociceptors can drive non-pain-like reflexive behaviors and pain-like affective behaviors *via* two distinct spinal pathways depending on physiological or pathological conditions, respectively. They suggest to focus on affective behaviors as better indicator of pain then reflexive behavior and emphasize to use the rational behavioral assays to measure pain.

Summarizing, this Research Topic presents recent important findings related to the role of various metals ions in the physiology and pathology of the central nervous system. It highlights the mechanisms of ion homeostasis dysregulation and links them to the development of various human pathologies, but also presents new perspectives for pharmacological interventions.

## Author contributions

All authors listed have made a substantial, direct, and intellectual contribution to the work and approved it for publication.

## Conflict of interest

The authors declare that the research was conducted in the absence of any commercial or financial relationships that could be construed as a potential conflict of interest.

## Publisher's note

All claims expressed in this article are solely those of the authors and do not necessarily represent those of their affiliated organizations, or those of the publisher, the editors and the reviewers. Any product that may be evaluated in this article, or claim that may be made by its manufacturer, is not guaranteed or endorsed by the publisher.

